# Learning the value of information and reward over time when solving exploration-exploitation problems

**DOI:** 10.1038/s41598-017-17237-w

**Published:** 2017-12-05

**Authors:** Irene Cogliati Dezza, Angela J. Yu, Axel Cleeremans, William Alexander

**Affiliations:** 1 Centre for Research in Cognition & Neurosciences (CRCN), Université Libre de Bruxelles, Brussels, Belgium; 2 Department of Experimental Psychology, Ghent University, Gent, Belgium; 3 Department of Cognitive Science, University of California San Diego, La Jolla, CA United States

## Abstract

To flexibly adapt to the demands of their environment, animals are constantly exposed to the conflict resulting from having to choose between predictably rewarding familiar options (exploitation) and risky novel options, the value of which essentially consists of obtaining new information about the space of possible rewards (exploration). Despite extensive research, the mechanisms that subtend the manner in which animals solve this exploitation-exploration dilemma are still poorly understood. Here, we investigate human decision-making in a gambling task in which the informational value of each trial and the reward potential were separately manipulated. To better characterize the mechanisms that underlined the observed behavioural choices, we introduce a computational model that augments the standard reward-based reinforcement learning formulation by associating a value to information. We find that both reward and information gained during learning influence the balance between exploitation and exploration, and that this influence was dependent on the reward context. Our results shed light on the mechanisms that underpin decision-making under uncertainty, and suggest new approaches for investigating the exploration-exploitation dilemma throughout the animal kingdom.

## Introduction

Imagine a nice summer day in Rome. You stop by a *gelateria* to buy a refreshing ice cream. After entering the shop, you are confronted with dozens of different ice-cream flavours. A good strategy would be to choose your favourite flavour (i.e., vanilla), because the likelihood that you will find it satisfying is very high. However, the shop also sells flavours that seem appealing but that you have never tasted (e.g., ginger and cinnamon). Do you select the flavour that you know you will enjoy, or do you select a flavour you have never tried before, potentially finding either a new favourite, or profound disappointment? The decision problem you are faced with is an example of the *exploration-exploitation* dilemma. It reflects a conflict between exploiting a known source of reward (i.e., vanilla) and exploring other less well-known sources (i.e., ginger and cinnamon) that may turn out to be either a more rewarding outcome, or a wasted opportunity.

Humans can resolve the exploration-exploitation dilemma through adopting different exploratory strategies, and different theoretical frameworks have offered different accounts of the factors that drive exploration. In reinforcement learning (RL) theory, exploration may be random: a decision-maker who learns to maximize a numerical reward signal^1^ may nevertheless make choices associated with lower reward value (exploration) due to a noisy response-generation process^2^. Alternatively, in optimal decision-making theories^3^, exploration is viewed as a directed or intentional process (i.e. directed or uncertainty-driven exploration). Here, the decision-maker is also trying to maximize reward, but he takes a longer view and is willing to sacrifice short-term high-reward choices so as to gain information about more uncertain novel options, ultimately hoping to achieve an overall higher level of reward^4^. In directed exploration, the *absence of information* related to the estimation of the reward value of an option constitutes a key factor for exploration: all else being equal (e.g., estimated reward value and reward variance of options), directed exploration minimizes the difference in information amongst options. On the contrary, random exploration is merely the result of a noisy process.

In previous research, evidence concerning the extent to which human decision-makers engage in directed exploration has been inconsistent. Some studies failed to observe directed exploration in humans^5–7^, possibly because of using tasks (i.e., “bandit tasks”) that make it difficult to separately identify directed and random exploratory strategies^8^ due to rise in information/reward confound^2^. To overcome this limitation, Wilson *et al*.^2^ recently developed a new version of the bandit task in which the information participants had about the payoffs of each slot machine was controlled, thereby eliminating the reward and information confound (i.e. information and reward were ‘decorrelated’). Based on this paradigm, Wilson *et al*. showed that participants indeed adopted both random and directed exploratory strategies: when participants were given unequal amounts of information regarding two options, they more frequently chose the option about which they had less information, even when that option was associated with lower gains.

Thus, withholding information about choice reward appears to promote directed exploration and to modulate the manner in which humans resolve the exploration-exploitation dilemma. However, in Wilson *et al*.’s task, payoffs concerning previous decision outcomes were displayed to participants at the time of the decision. In daily life, however, outcomes concerning previous choices are not always perfectly available; instead, it is their learned values that play the central role during the decision process. Moreover, previously learned outcomes influence future decisions in a way that depends on the type of learning involved^9^, on the context in which decisions are made^10^ and on the weight that each individual gives to decision outcomes^11^. Nonetheless, the number of times an outcome is experienced during learning might eventually influence the decision process. For example, increasing the number of times an option is observed decreases its uncertainty^3^, and the value of an option also depends on the level of uncertainty^12^. Accordingly, information might accumulate during learning, affecting the decision process itself. Consequently, learning information and reward over time might influence the resolution of the exploration-exploitation dilemma. Following this hypothesis, standard RL theories need to be augmented with optimal decision-making approaches specifying how reward and information interact to influence choices. Moreover, the context in which learning is achieved might influence this reward/information integration. Indeed, a vast literature has highlighted the crucial role of context in influencing decision strategies^10,13,14^. Experiencing richer contexts, for example, might speed up exploratory process as predicted by optimal foraging theory^15^.

**Figure 1:**
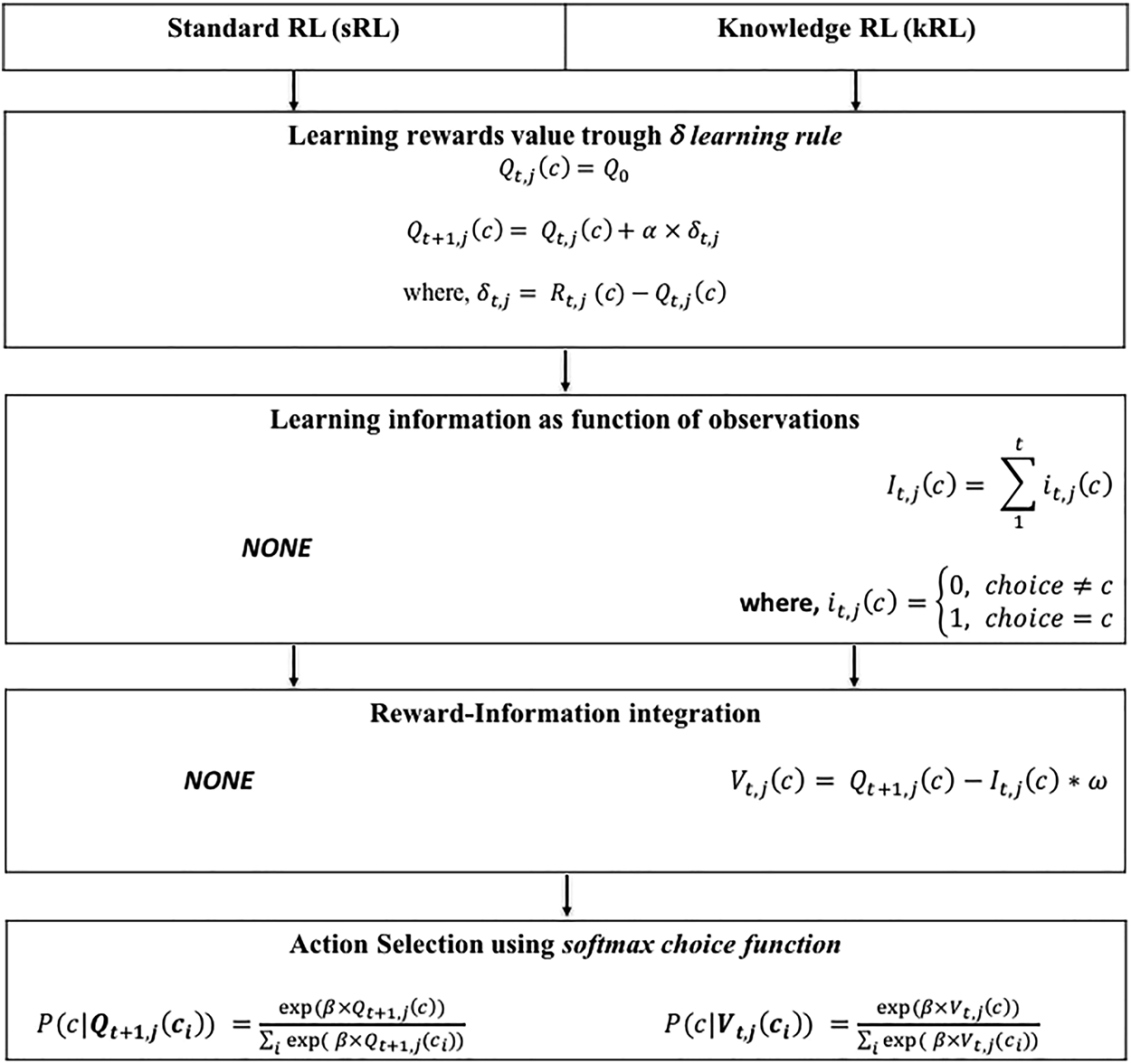
Comparison between sRL and kRL. Both models learn reward values *Q*_*t*+1_,_*j*_(*c*) during the forced-choice task using a δ learning rule, but only kRL learns also information ${I}_{t,j}(c)$- as function of the number of previous observations. At the end of the forced-choice task, kRL integrates information and reward in a same choice value *V*_*t*,*j*_(*c*) where the expected reward value *Q*_*t*+1_,_*j*_(*c*) is reduce by the amount of information gained during previous trials and its weighted parameter ω. In order to generate choice probabilities based on choice values, both models use a softmax choice function. However, sRL enters only expected reward values *Q*_*t*+1_,_*j*_(*c*), whereas kRL enters the combined information-reward value *V*_*t*,*j*_(*c*). The process is iterated for the length of free-choice task and at the beginning of each game expected reward values are initialized to *Q*_0_ and information to zero.

**Figure 2:**
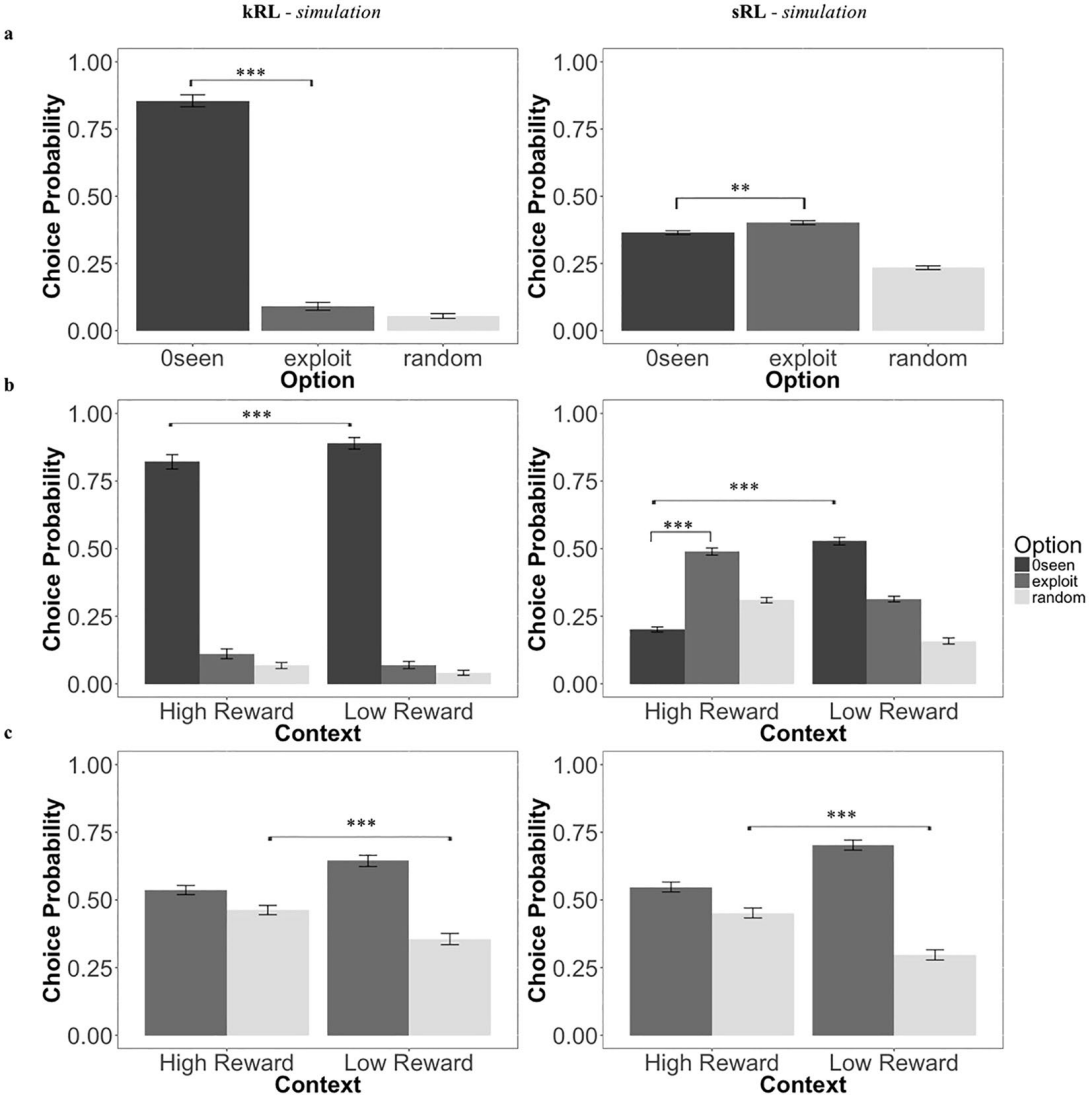
Model predictions. (**a**) In the unequal information condition (when information concerning the 3 decks was not always available), sRL (right frames) predicts a higher probability of choosing highly rewarded option (i.e., exploitation), whereas kRL (left frames) predicts a higher probability of choosing the most informative/never experienced option (i.e., 0seen). (**b**) Both models predict a decrement in directed exploration in high reward contexts (when the generative mean was set to 50 points) compared to low reward contexts (when the generative mean was set to 30 points). However, in the high reward context, kRL still chooses more often 0seen options compared to exploitation (left frames), whereas sRL chooses more often exploitative options (right). (**c**) In the equal information condition (Baseline + Reward) under different reward contexts, both models predict increased random exploration in the high reward context compared to the low reward context.

**Figure 3:**
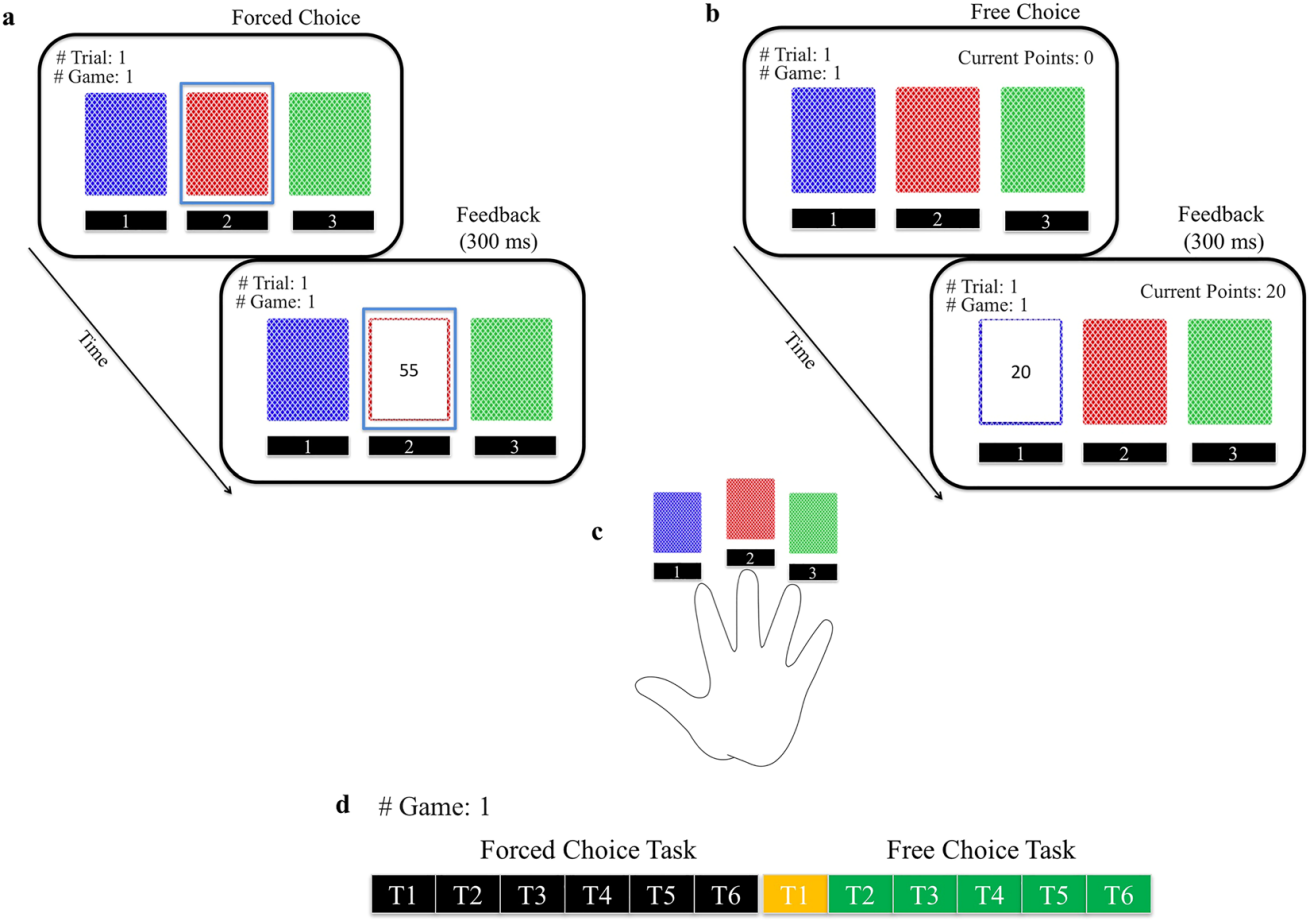
Behavioral paradigm. A variant of Horizon task^2^, in which participants chose between options in two different phases: *forced-choice task* and *free-choice task*. (**a**) During the forced-choice task, three decks of cards were displayed on the screen (a blue, a red and green deck) and participants were forced to choose a preselected deck outlined in blue. After selecting the deck, the card turned and revealed the points associated with the selected option, between 1 and 100 points. At this stage, the points displayed to participants were not added to their total score. To allow learning, feedbacks of previous trials did not remain visible to participants. The number of times each deck was played varied throughout the experiment: 2 times each deck - equal information condition; 0, 2, 4 times - unequal information condition. (**b**) During the free-choice task, participants made their own decisions among the same three decks of cards displayed during the forced-choice task. Participants were instructed to attempt to maximize the total points earned at the end of the experiment. After each trial, the points displayed on the screen were added to the participants’ total score. (**c**) Participants indicated their choices using the forefinger, middle finger and ring finger and pressing the keyboard keys ‘1’, ‘2’ and ‘3’, respectively. (**d**) During a game, participants faced 6 consecutive trials of the forced-choice task and between 1 and 6 trials of the free-choice task. The number of free-choice trials was exponentially distributed such that a higher proportion of games allowed subjects to make 6 free choices. Analysis in the Results section mostly focuses on choices made by participants during the first trial of the free-choice task (in yellow) being the only trial where reward and information were uncorrelated^2^.

In this study, we compare two possible computational solutions to the learning problem faced by humans during the exploration-exploitation dilemma: (1) a standard Reinforcement Learning (sRL) model where only rewards are considered during the learning and decision process; (2) and a novel reinforcement learning model in which quantities related both to reward and information are learned over time and combine to derive choice values (knowledge Reinforcement Learning- kRL; Fig. 1). Previous models^2^ have suggested how information and reward might be integrated in driving choice behaviour, but have not included trial-by-trial learning dynamics, thus potentially limiting the scope of their predictive power. Moreover, we extended previous measures of information^2^ to a dynamic and trial-dependent computation adaptable to more complex scenarios. Specifically, we computed information as discrete quantities, which accumulate over time as the number of observations of an option increases. Both learning models make different predictions regarding how humans solve the exploration and exploitation dilemma when information concerning competing options is not always available (e.g., not integrating information tends to facilitate exploitative strategies), and when it is presented under different learning contexts (e.g., higher reward context reduces exploration of most informative options in a way that differs when the algorithm integrates or not integrates information) (Fig. 2). To assess the models’ predictions, we developed a novel version of the Wilson *et al*. paradigm in which participants make repeated choices about three decks of cards in such a way that previous deck-selection influences choices through learning (i.e., outcomes disappeared from the screen after 300 ms of deck selection - Fig. 3). The use of three options allows us to investigate participants’ behaviour in a more complex and realistic scenario. By comparing the fits of the two models to trial-by-trial participant responses, our aim was to establish which model better describes participants’ behaviour while performing our task. In the following sections, we present the main results whereas the mathematical description of the two models and their predictions, derived from model simulations, can be found in the last section of this manuscript.

## Results

In this section, we first describe the analysis related to participants’ behaviour investigating the presence of behavioural patterns in participants’ data predicted by each model (qualitative model comparison analysis). Subsequently, we present a quantitative analysis to investigate which model better explained participants’ choices. Because all participants accurately performed the gambling-task, playing strategically and learning reward outcomes throughout the experiment (for details refers to supplementary material), we did not exclude any participants from our analyses.

### Qualitative model comparison analysis

#### Prediction 1: Directed Exploration vs. Exploitation

**Figure 4:**
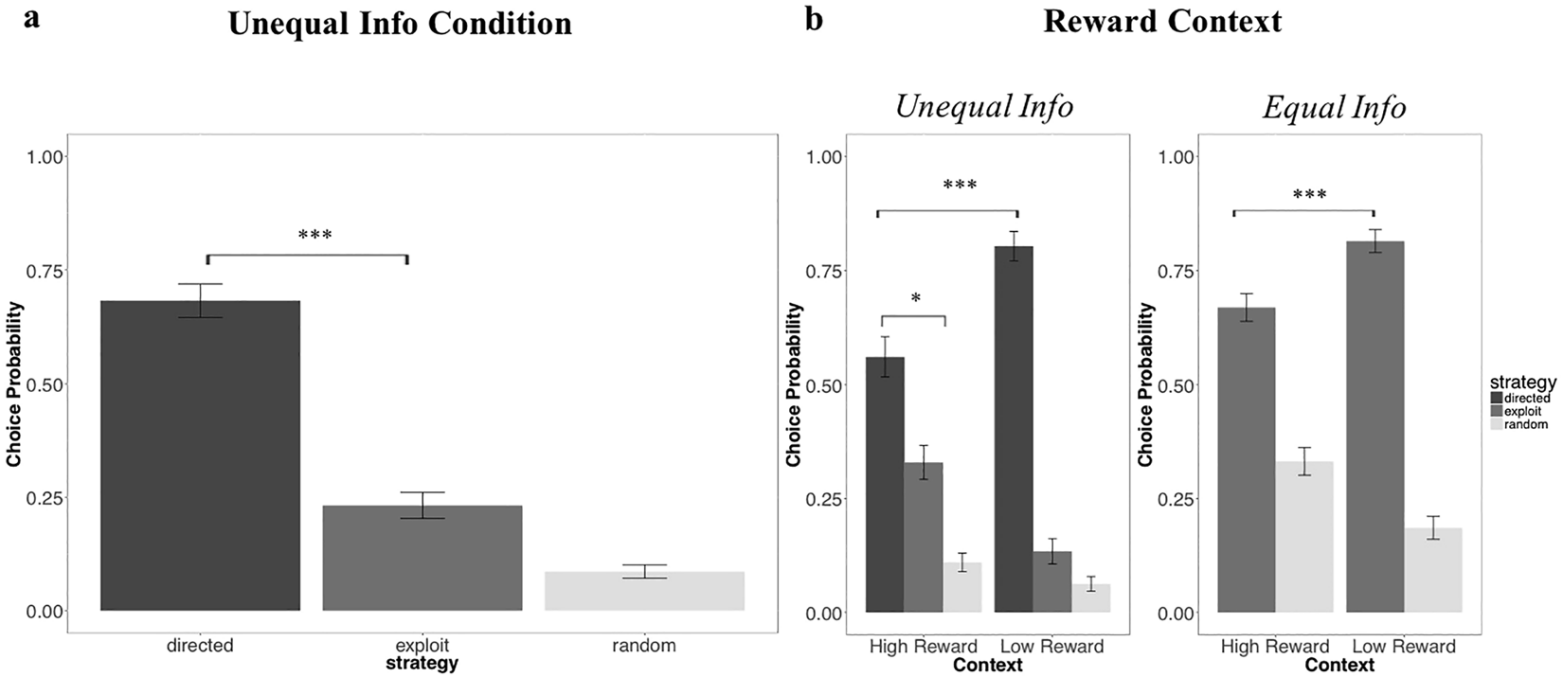
Human behaviour. (**a**) When information concerning the 3 decks was not always available – unequal information – participants were more likely to directed explore in the first trial of the free-choice task compare to exploit and random explore as predicted by kRL. The graph reports results related with Information condition only (when mean reward were equals among the decks). (**b**) When unequal amounts of information were available from the decks (left), the probability of exploitation and random exploration increases under the High Reward context while the probability to directed explore decreases as predicted by both models. However, participants showed higher preferences toward directed exploratory options compared to exploitative ones as predicted by kRL. In the equal information condition under different reward contexts (right), participants were more likely to engage in random exploration in the High Reward context and to engage in exploitation in the Low Reward context as predicted by both RL models.

The first prediction of kRL and sRL concerns the information manipulation condition, in which decks are sampled unevenly during the forced-choice task (Fig. 2a). Under this scenario, the kRL model predicts more frequent selection of the never-experienced deck (0seen) whereas sRL predicts to more frequent selection of options associated with highest expected reward (exploitation). To investigate how information manipulation affected participants’ strategy selection, we computed directed exploration, random exploration and exploitation in the first free choice trials of the Information condition (where information was manipulated but mean reward was equal among decks; see Methods). We conducted the analysis on the first trial of each game, it being the only trial where reward and information confound were uncorrelated^2^. Trials were classified as directed exploratory when participants chose the option that was never selected during forced-choice trials (0seen deck), exploitative when participants chose the experienced deck with the highest average of points (regardless of the number of times that deck had been selected during the forced-choice task) and random exploratory when the classification did not meet the previous criteria. Averaged values were entered into single factor repeated measure ANOVA. Results revealed an effect of information on decision strategies F(1,40) = 80.65, *p* < 10^−14^ (Fig. 4a). Pairwise comparison using paired t-test showed significant differences between all comparisons with all *p* values <10^−4^, indicating that under the manipulation of information participants adopted a significantly more directed-exploratory strategy (M = 0.682, SD = 0.168) to the detriment of random exploration (M = 0.086, SD = 0.1069) and exploitation (M = 0.232, SD = 0.132). This analysis is in line with the first kRL prediction where the probability of engaging in directed exploration is higher compared to exploitation (for details refer to method section). Similar results were obtained investigating the decision strategies overall in the unequal information condition (Information + Mixed condition) and in the Mixed condition alone (where both information and reward were manipulated- see supplementary material).

#### Prediction 2: reward context

The second prediction concerns the effect of reward context on decision strategies (Fig. 2b). Both the sRL and kRL models predict reward context-dependent changes in selecting the 0seen deck (for details see method section). To investigate the models’ predictions, we computed random exploration and exploitation in the first free choice trials of the Information condition under High and Low Reward context. We conducted a 2 (context: Low Reward, High Reward) by 3 (strategies: exploitation, random exploration, directed exploration) non-parametric ANOVA. The test allows the use of two-way repeated measure ANOVA in a non-parametric setting using aligned rank transformation (e.g., ARTool package in R, http://depts.washington.edu/madlab/proj/art/)^16^. Results showed an effect of strategy F(2,100) = 161.9, p < 10^−15^, and an effect of context X strategy F(2,100) = 26.359, p < 10^−9^ (Fig. 4b), whereas we did not find a general effect of context, *p* = 0.926. Post-hoc comparisons showed an increase in exploitation in High Reward context (M = 0.329, SD = 0.171) compared to the Low Reward (M = 0.134, SD = 0.128), *p* < 10^−5^; a decrease in directed exploration in the High Reward context (M = 0.561, SD = 0.201) compared to the Low Reward context (M = 0.803, SD = 0.149), *p* < 10^−7^; and an increment in random exploration in the High Reward context (M = 0.11, SD = 0.092) compared to the Low Reward context (M = 0.062, SD = 0.074), *p* = 0.042. The above results are in line with both models’ predictions where the probability to be engaged in directed exploration was reduced in High Reward context. However, the two models draw different predictions concerning the degree by which the 0seen deck is selected. As a result of information integration in choice value, kRL predicts higher probability of selecting the 0seen deck in both reward contexts, whereas sRL predicts to select most often exploitative options in High Reward context. Post-hoc comparisons in the High Reward context showed that participants performed directed exploration significantly more compared to exploitation, *p* = 0.0104 as predicted by the kRL model.

To examine the models’ predictions in the equal information condition (both models predict an increase in random exploration in the High Reward context- Fig. 2c), we computed decision strategies under High and Low Reward context. Here, the first trial of each game was labelled as exploitation when participants selected the deck with the highest average number of points gained during the forced-choice task, and as random exploration otherwise. A 2 (context: Low Reward, High Reward) by 2 (strategy: exploitation, random exploration) non-parametric ANOVA was adopted. The results showed an effect of strategy F(1,60) = 216.4, *p* < 10^−15^, and effect of condition X strategy F(1,60) = 26.9, *p* < 10^−5^) (Fig. 4b), whereas a general effect of context did not reach significance, *p* = 1. Post-hoc comparison indicated an increment in random exploration in High Reward (M = 0.331, SD = 0.139) compared to Low Reward context (M = 0.186, SD = 0.115), *p* < 10^−4^ as predicted by both sRL and kRL model. In the supplementary material, we show that this increase in random exploration in the High Reward context might be due to an effect of utility vs. cost induced by reward context (Fig. S2).

#### Horizon, Uncertainty Preference, and Directed Exploration

**Figure 5:**
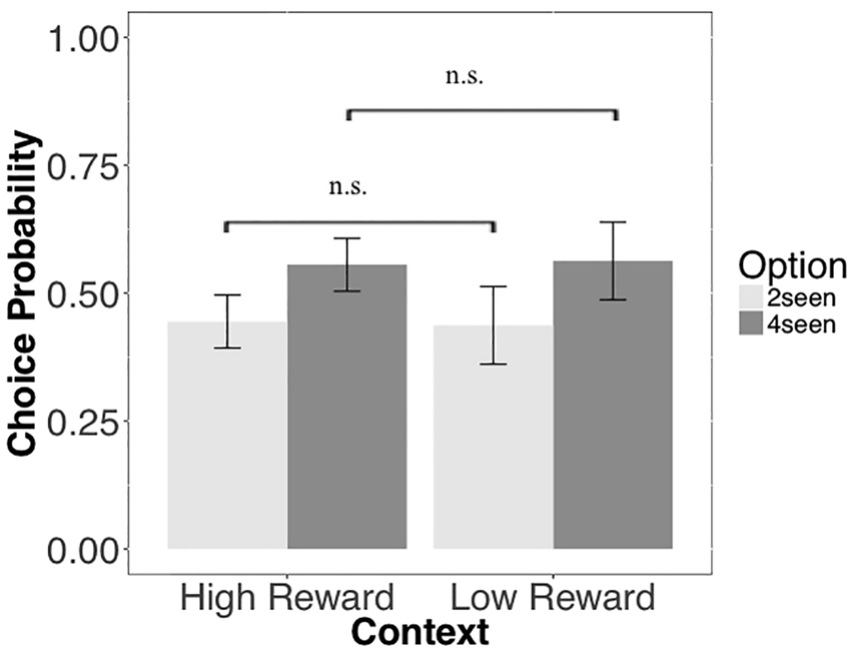
Uncertainty preference and ‘seen analysis’. Probability to choose in the first free-choice trials the option experienced 2 times (2seen) and 4 times (4seen) during the forced-choice task under different reward conditions. 2seen and 4seen decks were chosen at the same rate within and between reward conditions suggesting that choices labelled as directed exploration where indeed guided by an exploratory drive and not by participants’ preference toward uncertainty.

In Wilson *et al*.^2^, directed exploration was quantified as an increase in exploratory decisions on the first free-choice trial for conditions in which participants were allowed multiple free choices vs. conditions in which they were allowed a single choice. In our version of the task, we do not include a specific horizon manipulation: the number of free-choice trials was selected randomly from an exponential distribution such that, in a plurality of games, subjects were allowed 6 free-choices. However, subjects were not informed as to the number of free-choice trials they would experience during a game. Instead, in order to disentangle directed exploration from random exploration, we used a version of the task in which subjects were faced with 3 options. Given our version of the task did not include a specific horizon manipulation, it is possible that our interpretation of choices of the 0seen option as evidence of directed exploration may be confounded with “uncertainty preference”^17^. As discussed in that study, uncertainty preference arises when, in choosing the least observed option, the potential use of future information is irrelevant (in contrast with directed exploration where potential use of future information is the driving factor of information seeking). These authors observed an increase in uncertainty preference in a loss context compared to gain context^17^. However, our use of 3 options allows us to rule out uncertainty preference as the sole explanation for the observed decrease in selection of the 0seen option in high reward contexts relative to low reward contexts. Because uncertainty preference should act on both 0seen and 2seen options, if uncertainty preference was the driving factor in our task, we should also expect to find higher selection of the 2seen option relative to the 4seen option during the Low Reward context compared to the High Reward context. We therefore tested this by computing the probability of choosing each of the non-zero options (2seen and 4seen) in High and Low Reward context of the Information condition. A 2 (option: 2seen, 4seen) by 2 (context: High Reward, Low Reward) repeated measure ANOVA did not show any effect of option *p* = 0.289, any effect of context *p* = 0.584, or interaction effect between context and option *p* = 0.919, ruling out uncertainty preference as a factor underlying differences in choice behaviour between high and low reward contexts (Fig. 5).

### Quantitative model comparison analysis

**Figure 6:**
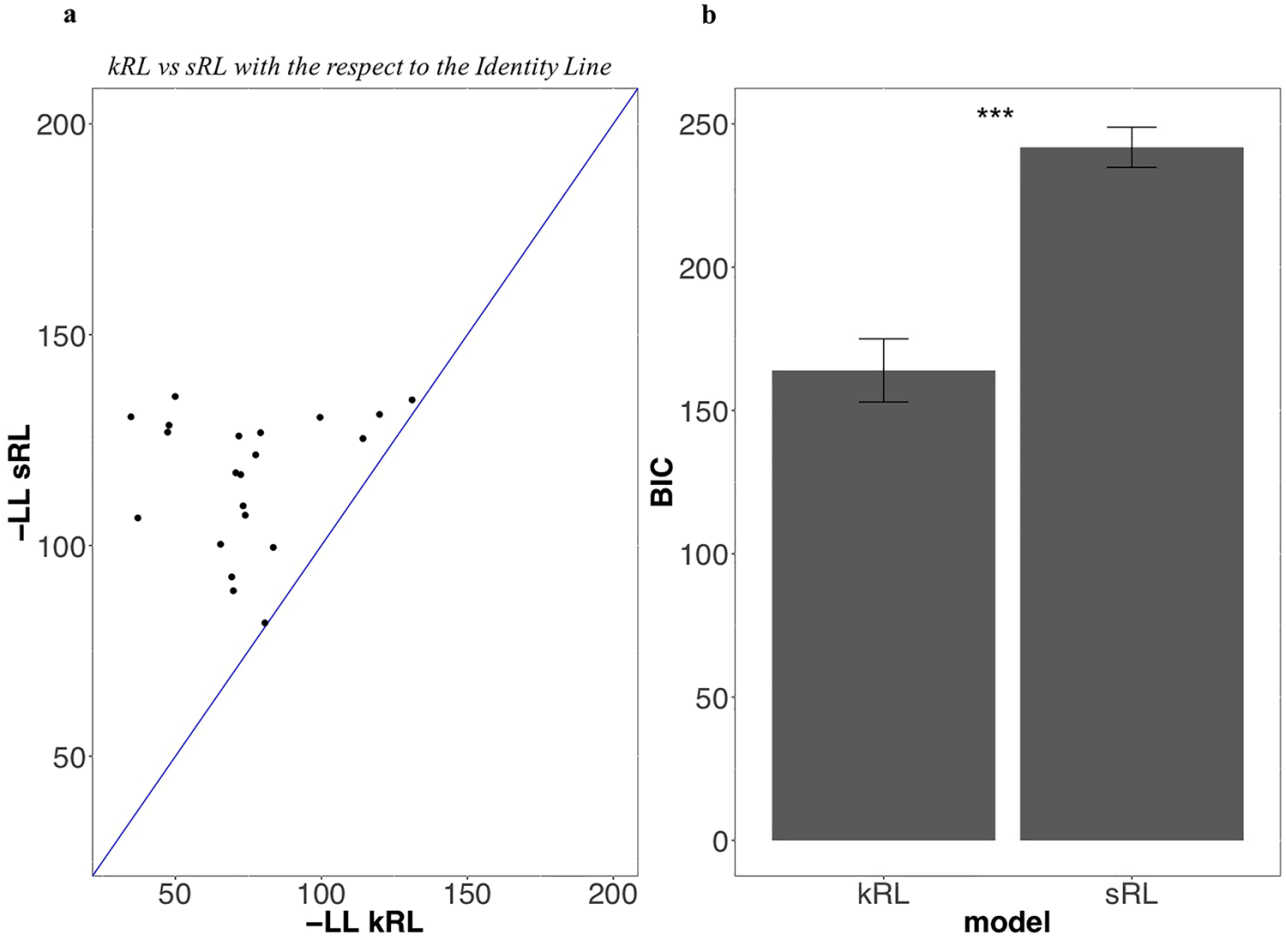
Comparative fit of the kRL and sRL. (**a**) The comparison of the fit is based on the negative log likelihood (i.e., probability of observing the data given the model) of both models. Each point is one participant. The kRL fits better when the point is below the 45° line. Except for one participant who is lying on the identity line, all participants’ behaviour is better represented by kRL model. (**b**) BIC of both models (i.e., the likelihoods of the data given the model while accounting for their different numbers of free parameters). kRL had a significantly lower BIC compared to sRL. Model with lower BIC values are better able to explain the variability in the data.

Our analyses of behavioural data provide support for the kRL account of participants’ behaviour. We additionally conducted a quantitative model comparison analysis to investigate which model best predicted participants’ decision process. We first investigated the goodness-of-fit by comparing kRL and sRL model responses to participants’ responses toward 0seen options in the Information condition. R-squared computation revealed that kRL was able to explain approximately the 90% of variance in participants’ data, R^2^ (19) = 0.8975 *p* < 10^−11^, whereas sRL explained approximately 10%, R^2^ (19) = 0.073 *p* = 0.236. Subsequently, we graphically investigated the two models in terms of negative log likelihood. Only one participant did not benefit from one of the two models laying on the identity line (Fig. 6a). However, because R-squared and likelihood tend to favour complex models (i.e., models with more explanatory variables) we also compared the likelihoods of the data given the model while accounting for their different numbers of free parameters (i.e., model complexity). To do so, we computed BIC and AIC^18,19^ values as reported in the methods section.

We conducted a classical frequentist analysis with BIC values of the two models entered into the Wilcoxon Signed Rank Test. Results showed that kRL model (BIC_kRL_ = 163.978) best represented participants’ data compared to sRL (BIC_sRL_ = 241.838), Z = 3.945, p < 10^−6^ (Fig. 6b). Results for AIC values were similar to those for BIC values (Table 1). Fitting to all free-choice trials did not change model comparison results, and simulating both models with the best-fit parameters reproduced behavioural patterns observed during simulation with random parameters (details for both analysis in the supplementary). In sum, both our qualitative and quantitative model analyses indicate that participants’ choice behaviour integrated both reward and information into a choice value.

**Table 1:** Model Comparison.

	sRL (1^st^choices)	kRL (1^st^choices)	gkRL (1^st^choices)	sRL (All Trials)	kRL (All Trials)	gkRL (All Trials)
BIC	241.838 (32.1)	163.978 (50.603)	163.141 (52.469)	809.064 (98.152)	791.092 (97.723)	683.505 (143.789)
AIC	236.135 (32.099)	155.424 (50.603)	151.736 (52.47)	800.554 (98.153)	778.326 (97.724)	666.484 (144.791)
Neg. Log-Lik	116.068 (16.05)	74.712 (25.302)	71.868 (26.235)	398.277 (49.077)	386.163 (48.862)	329.242 (72.395)

BIC, AIC, negative log-likelihood group average and standard deviation reported for sRL, kRL, and gkRL models fitted to both the 1^st^ and all free-choice trials. Models that best scored during the model comparison are underlined.

#### Non-linear information gain

Although kRL was better able to explain the participants’ behaviour using a parametric integration of information, our assumption, building from the binary coding of information suggested by Wilson *et al*.^2^, that information scales linearly with the number of samples (equation 4) may be incorrect. It may be the case that information gain is subject to diminishing returns: each additional observation of an option may yield progressively less additional information regarding that option. Alternately, it may be the case that, at least for situations (as in the present task) in which only a few samples of each option are available, additional observations may provide a greater-than-linear amount of information. To better investigate the nature of the scaling, we evaluated the kRL model against a version (the gamma kRL, or gkRL model) that allows information to increase sub- or super-linearly with the number of observations:

1 $${I}_{t,j}(c)={(\sum _{1}^{t}{i}_{t,j}(c))}^{\gamma }\mathrm{where},{i}_{t,j}(c)=\{\begin{array}{c}0,\,choice\ne c\\ 1,\,choice=c\end{array}$$

Here information was computed by including an exponential term, gamma, for the sum of observations *I*_*t*,*j*_(*c*) and then integrating this computation in the choice value *V*_*t*,*j*_(*c*) as in equation (5). Gamma defines the degree of non-linearity in the amount of information obtained as a function of the number of observations, *i*_*t*,*j*_ Choices were made as in equation 6. As with the kRL model, we fit the gkRL model to both first free-choice trials and all free-choice trials and conducted model comparisons. A paired t-test on BIC values showed no difference between gkRL model (BIC_gkRL_ = 163.141) and kRL (BIC_kRL_ = 163.978), t(20) = −0.595, *p* = 0.559, when fit to first-free choices only, whereas a paired t-test on AIC values revealed a lower value in gkRL model (AIC_gkRL_ = 151.736) and kRL (AIC_kRL_ = 155.424), t(20) = −2.62, *p* = 0.016. However, when fit to all free-choices, Wilcoxon Signed Rank Test showed gkRL model (BIC_gkRL_ = 683.505) best represented participants’ data compared to kRL (BIC_kRL_ = 791.092), Z = 6.054, p < 10^−4^. The discordant results obtained when fitting only the first free-choice trials might be due to the bias toward simple models for BIC and to complex models for AIC^20^. While the gkRL model was not decisively better than the kRL model when fitted to first free-choice trials, the BIC and AIC scores from all trials suggest at least equivocal evidence in favour of the gkRL model. Analysis of the gamma parameter estimates suggest that, to the extent the gkRL model explains our data better than the kRL model, it is due to a sub-linear influence of observations on information. The mean gamma parameter estimate when fit only to the first free-choice trials was 0.411, significantly lower than 1 (linear), p < 10^−4^. This sub-linear influence is more pronounced in fits of the gkRL model to all free-choice trials: here the mean gamma estimate is 0.103, significantly lower than the estimated gamma parameter for fits only to the first free-choice trials (p = 0.002). These results suggest that, as the number observations increases (in the free-choice trials), the sub-linear information gain becomes more relevant, while in the first free-choice trial, the effective level of information gained from 4 observations, while less than double that gained from 2 observations, is still behaviourally relevant.

## Discussion

The exploration-exploitation dilemma is a fundamental conflict faced by humans when adapting to the demands of their environment. The resolution of the dilemma is achieved following either a random or a directed strategy. However, in previous work directed strategies were mostly confounded by random strategies making difficult to investigate whether information processing influenced the resolution of the dilemma. In this study, we implemented a version of a previous paradigm that is able to separately identify directed and random strategies^2^ and we examined how learning about reward and information over time influences the resolution of dilemma. We modified the previous version of the paradigm to make learning a requisite for good performance (see also^21^), and we compare two computational solutions to investigate the learning problem. Our results suggest that humans learn a combined reward/information value when facing sequential exploration-exploitation problems. Moreover, the degree to which reward and information are integrated depends on the learning context in which decisions are made (i.e., the number of times options are selected and the overall level of reward associated with the options). Together, these findings shed additional light on the mechanisms underlying decision-making under uncertainty and suggest new approaches for investigating the exploration-exploitation dilemma. In the following, we discuss the implications of our main results.

Our data showed that participants played strategically during the games, learning about reward outcomes throughout each game. Moreover, participant behaviour was better described by a δ learning rule compared to estimating an average of observed outcomes (supplementary material), as suggested by previous works^2^. Furthermore, our results showed that choices made by participants reflected the integration of both reward and information over time, replicating findings that information-seeking behaviour intervenes during the resolution of the exploration-exploitation dilemma^2,22,23^. When making decisions among alternatives that differ in their informative value, participants preferentially sample from options that will provide more information, inhibiting their default tendency to choose highly rewarded options^5^ in favour of more uncertain, but potentially better, alternatives. Under previous formulation, information gain is prospective, and prospective gains in information contribute positive value to the expected value (in points) of choosing an uncertain option^2^. Conversely, in kRL’s formulation, information is considered retrospective - the amount of information acquired during the past- and it is added as negative value: directed exploration is realized by reducing the probability of selecting well-known alternatives. Our results showed that participants integrated information as parametric function of the number of observations of one option compared to a simple binary coding scheme signifying greater or lesser information (supplementary material) as previous models would predict^2^. Our results also suggest that information integration is sub-linearly related to number of observations, consistent with decreases in uncertainty in the Bayesian posterior (quantified by either entropy or variance). While both retrospective and prospective formulations can account for exploratory behaviours in humans, the exact manner in which information is computed and is integrated in choice values remains an open question and should be the subject of future research.

While our study replicates prior findings of the use of information during the resolution of the dilemma, the evidence concerning directed exploration has is not been consistent in the literature. Several studies have failed to find information integration during exploration^5,6^. For example, in Daw *et al*.^5^, a model that considered only reward as driving factor for its choices was better able to explain participants’ performance on a bandit task compared to a model that also integrated information. However, the particular instantiation of bandit tasks in those studies might explain this failure^8^. Generally, in bandit tasks, reward and information are confounded^24^, hence making it difficult to discern random from directed exploration^2^. Additionally, bandit tasks often involve drifts in the rate of payoffs associated with each bandit^5^, leading to the possibility that participants cannot accurately track reward mean or variance as predicted by the model. Therefore, even if subjects did utilize directed exploration, the model would not be able to uncover it due to mis-modeling of subjects’ probabilistic beliefs and the reward distributions.

Additionally, our data indicated that the reward context (i.e., the overall level of reward associated with the options) may influence how humans integrate reward and information when solving the exploration-exploitation dilemma. In higher reward contexts, participants showed an increment in their preference for highly rewarded decks and a decrement in their preference for the most informative option. This effect was not due to a context-dependent decrease in uncertainty preference, but it was instead a specific effect of directed exploration. These results are in line with previous findings where learning contexts appeared to influence decision-making strategies^13,14^. Moreover, the framing effect^10^ can explain the decrement in directed exploration observed in our study during high reward context: when experiencing gains, humans tend to engage in uncertainty-averse behaviours^17,25^. Furthermore, framing the dilemma in terms of losses has also been associated with exploratory strategies^26^. Exploratory behaviour thus seems to depend both on the amount of information regarding available options as well as the context in which the decision is made. However, the results of our study concerning random exploration nuance this view; we found that random exploration increased under high reward context both in the equal and unequal information conditions. Our results suggest that the decrease in random exploration in low reward context might be explained by an increase in cost or by a decrease in utility of switching among alternatives in this condition (supplementary material). These findings are in line with predictions made by optimal foraging theory^15^: in a rich habitat, exploration among patches may increase because searching further is more likely to yield better outcomes, whereas in poorer environments, patch exploitation increases because it will be more difficult and costly to find better outcomes^27^. Thus, two different processes appear to underlie the resolution of the exploration-exploitation dilemma under different reward contexts, and their characterization depends on the type of exploratory strategies adopted. Our results are in line with recent findings where random and directed exploration showed distinct underlying mechanisms^21^, however our results also confirm that both random and directed exploration show a common exploratory drive (see supplementary).

Although our study replicated findings of the use of information by humans during the resolution of the dilemma bringing additional knowledge on the learning mechanism involved, there are nonetheless limitations that may influence the scope of our results. These limitations relate primarily to the formulation of our task as a series of discrete games in which task-relevant information (global mean reward value, information about options) is reset prior to the start of each game. In typical bandit tasks, which have been well-studied from a cognitive and machine-learning perspective, the value of options evolves over the course of an experiment, leading to the incorporation of mechanisms by which the influence of previous observations is discounted as a function of time^28^, estimates of uncertainty related to ongoing experience with the task^5,22^, and continuous adjustments of value predictions for options. In order to adapt the kRL model to these more traditional tasks, similar mechanisms may need to be included in future iterations of the model.

Despite these limitations, a new view of the exploration-exploitation dilemma is suggested, considering exploration and exploitation trade-off as a class of problems spanning different scales^29,30^. We showed that depending on the scale adopted (information, reward, experience, context) the resolution of the dilemma might differ as well as its hidden mechanisms. Future studies should take into account this perspective to better explain exploration-exploitation phenomenon throughout the animal kingdom.

## Methods

### Participants

Twenty-one young healthy adults participated in this study (12 women; aged 19–29 years, mean age = 23.24). The inclusion criteria were as follows: normal color vision, right-handed, and absence of psychoactive treatment. The entire group belonged to the Belgian Flemish-speaking community. The experiment was approved by the UZGent ethics committee and conducted according to the Declaration of Helsinki. Informed consent was obtained from all participants prior to the experiment.

### Procedure

Participants played 128 games of a gambling-task, where on each game repeated choices were made among 3 decks of cards (Fig. 3). The task is a variant of the Horizon task^2^, in which participants chose between two options in two different phases: *forced-choice task* and *free-choice task*. Our experimental task differs in four ways from the Wilson *et al*. task. First, participants chose among three options, depicted as decks of cards (Fig. 3). Second, information regarding the points earned following a choice did not remain visible following feedback, allowing learning during the task. Third, while the forced-choice task lasted 6 trials in all the games (constant number as in Wilson *et al*.^2^), the length of the free-choice task ranged from 1 to 6 trials. The number of free-choice trials was exponentially distributed such that a higher proportion of games allowed subjects to make 6 free choices. Fourth, the length of the free-choice task (i.e., horizon) was not cued to participants, so that in our version participants were not aware of the total number of free-choice trials they would have on any trial.

The decks of cards were arranged on the left, centre and right side of the computer screen (Fig. 3) and participants indicated their choices using the keyboard keys ‘1’, ‘2’ and ‘3’, respectively. After each choice, the chosen card was flipped to reveal a number indicating the points earned by the participant for selecting that deck. Each option paid off between 1 and 100 points, and the number of points earned for selecting a deck was sampled from a Gaussian distribution with standard deviation of 8 points. On each game, the generative mean of each deck was set to 30 (Low Reward condition) or 50 (High Reward condition). To avoid the influence of learned meta-information regarding the task (specifically, the possibility that participants may learn that, on each game, the points for each deck are generated from a specific mean of determinable value, and thus could estimate the value of a deck after a single trial), deck means were additionally adjusted by +/− 0, 4, 12, & 20 points on each game (generative Gaussian ranges from 10 to 70 points). The mean and standard deviation of the generative Gaussian were stable within a game and varied between games. The 3 decks of cards had the same generative means in 50% of the games (*equal reward*) and different means in the rest of the games (*unequal reward*). In the unequal reward condition, the generative means differed so that two options had higher means compared to the third one in 25% of the total games (High Reward Context) and had lower means in the remaining games of the unequal reward condition (Low Reward Context).

The use of three options allows us to dissociate effects related to directed exploration from random exploration between different reward contexts. For example, if choices that maximize information gain decrease from one context to another, this decrease could be a consequence either of *decreased* directed exploration (participants become less information seeker), or *increased* random exploration (participants’ choices become noisier). If the change is due to an increase in random exploration, then, for trials in which the subject chose one of the remaining available options in which information gain is not maximized, the probabilities of the subject choosing either option should be closer together and around chance. Conversely, if the change in information maximization is due to decreased directed exploration, thus not affecting the level of noise in participants’ choices, the probabilities of choosing the other two options should not change from one context to another.

During the forced-choice task, we manipulated the information about the decks of cards acquired by participants (i.e., the number of times each deck of cards was played). The information manipulation we adopted differed from Wilson *et al*. Here, on each game, participants were forced to either choose each deck 2 times (*equal information condition*), or to choose one deck 4 times, another deck 2 times, and 0 times for the remaining deck (*unequal information condition*). We decided to adopt this manipulation so as to maintain the differences of information among decks (4 times – 2 times and 2 times – 0 times) to be two, as in Wilson *et al*. In 50% of the games, participants played with the equal information condition. The order of card selection was randomized in both information conditions.

Participants were instructed to choose among the three decks of cards in the free-choice task so as to maximize their final score (i.e., the amount of points earned throughout the experiment). The total score was converted to a monetary payoff at the end of the experiment (i.e., 0.01 euros every 60 points). Participants were told that during the forced-choice task of certain games they could sample options at different rate, and that the decks of cards did not change during the same game, but were replaced by new decks at the beginning of each new game. However, they were not informed of the reward manipulation and the underlying generative distribution we adopted.

Crossing the information and reward manipulations, we therefore obtained 4 different conditions: *Reward*, *Information*, *Baseline*, and *Mixed*. Reward and information were the main conditions, whereas Baseline and Mixed were the control conditions. The main conditions were used to test our hypotheses. Each of the main conditions consisted of 42 games each. In the Reward condition, reward was manipulated (*unequal reward*), whereas information was equal (*equal information*). In the Information condition the generative means of each option were equal (*equal reward*) and information varied (*unequal information*). The control conditions consisted of 20 Baseline games and 22 Mixed games. In the Baseline condition, both reward and information for each deck were equal (i.e. the generative means and information were equal among the 3 decks, and each deck was seen twice during the forced-choice phase), whereas in the Mixed condition both manipulations were introduced (*unequal reward/unequal information*). In each condition, reward context was manipulated by setting the mean of the Gaussian to 30 in 50% of the games and to 50 in the rest of the games to which the noise of +/− 0, 4, 12, 20 points per deck was added for each specific game. For each participant, the order of appearance of the 4 conditions was selected randomly, so each participant experienced a different game sequence.

### Computational models

To achieve good performance during our task, participants needed to learn reward outcomes associated with each deck of cards on a trial-by-trial basis. Tracking this information helps them maximize their final score and earn more money during the experiment. Because our task involved learning reward outcomes over time, we implemented two computational models based on reinforcement learning. In order to quantify how reward and information may influence choice behaviour, we compared two alternative RL models to determine which model could potentially explain participants’ behaviour during our task. The first model is a standard Reinforcement Learning model (sRL) that focuses its learning on reward outcomes only, and uses reward expectations as the driving factor of choice. The second model is a knowledge Reinforcement Learning that learns reward outcomes (as in sRL), and tracks the information gained from experienced decks. At the time of the decision, kRL integrates reward and information into an overall choice value. A comparison between the features of sRL and kRL is shown in Fig. 1. In the following sections, we present the mathematical description of the two models as well as their predictions, derived from model simulations.

#### Standard RL model

The sRL model uses a simple δ learning rule^31^ to compute the expected reward value *Q*(*c*) for each deck of cards *c* (=Left, Central or Right) on each trial, using the following equation:

2 $${Q}_{t+1,j}(c)={Q}_{t,j}(c)+\alpha \,\times {\delta }_{t,j}$$

where *Q*_*t*,*j*_(*c*) is the expected reward value for trial *t* and game *j*. ${\delta }_{t,j}=\,{R}_{t,j}(c)-{Q}_{t,j}(c)\,\,$is the *prediction error*, which quantifies the discrepancy between the previous predicted outcome and the actual outcome obtained at trial *t* and game *j*. The prediction error enables the model to adjust its predictions on a trial-by-trial basis, adapting reward expectations to new information. The degree by which new outcomes are integrated with the expected reward value *Q*_*t*+1_,_*j*_(*c*) depends on the learning rate *α*. With small *α* the model slowly updates it estimate of Q in response to new information (i.e., points obtained after a trial), whereas with higher values the model integrates new information more rapidly. In other words, with lower learning rate the model expects lower rate of change of the environment^32^. The expected reward *Q*_*t*+1_,_*j*_(*c*) is updated using the above rule only if an outcome from the deck *c* is observed, otherwise *Q*_*t*+1_,_*j*_(*c*) keeps the value of the previous trial. In order to generate choice probabilities based on expected reward values, the model uses a softmax choice function^5,33,34^. The softmax rule is expressed as:

3 $$P(c|{Q}_{t+1,j}({c}_{i}))\,=\frac{\exp (\beta \times {Q}_{t+1,j}(c))}{{\sum }_{i}\exp \,(\exp \,\beta \times {Q}_{t+1,j}({c}_{i}))}$$

where *β* is a free parameter known in reinforcement learning as the inverse temperature. *β* determines the degree to which choices are directed toward the highest rewarded option. With higher *β* the model is mainly exploitative, whereas with lower *β* the model chooses the deck more randomly. Considering that participants were told that games were independent from one another, *Q*_0_ is initialized at the beginning of each game^35^. However, despite the independence among games, we assumed that participants could learn the long-run average of all decks over the course of the experiment; that is, participants did not begin each game as if starting the experiment with no knowledge, but with an estimate of the possible average value of each deck. In other words, prior reward expectations *Q*_0_ are set as the value of the mean between the two-main generative Gaussian distributions. To obtain a global estimate of the expected reward values for each deck, we simulated the δ learning rule on 1200 forced-trials ×128 games. For each game, Q values were initialized to 0 and updated over all 1200 trials. The final Q values for each game were recorded, and the average of the final Q values (~40 points for each option) for each of the 128 games were used as the value of *Q*_0_ (equation 2).

#### Knowledge RL model

To account for potential influence of information on choice values, we implemented a version of the sRL that incorporates a mechanism reflecting the knowledge gained about each deck during a game - the kRL model. As in the sRL, the kRL model computes expected reward values using the δ learning rule described in equation 1, and *Q*_0_is initialized using the global estimate of reward values (*Q*_0_). Additionally, kRL tracks information gained from each deck based on how often it is selected, as follows:

4 $$\begin{array}{cc}{I}_{t,j}(c) & =\sum _{{\rm{l}}}^{t}{I}_{t,j}(c)\\ {\rm{w}}{\rm{h}}{\rm{e}}{\rm{r}}{\rm{e}},\quad {I}_{t,j}(c) & =\{\begin{array}{cc}0, & choice\ne c\\ 1, & choice-c\end{array}\end{array}$$

*I*_*t*,*j*_(*c*) is the amount of information associated with the deck *c* at trial t and game j. After six trial of forced choice task, if one option has never been selected *I*_*t*,*j*_(*c*) has value zero, whereas in the case one option is selected 4 times, *I*_*t*,*j*_(*c*) has the value 4. This dynamic and trial-dependent measure of information has been developed as an extension of the previous ‘nothing-or-all’ information bonus discussed in^2^. As in previous work^2^, information is assumed to interact with reward in influencing choice behaviour as an additional factor in determining value, and this interaction may positive (more information regarding a deck increases the subjective value of that deck) or negative (more information regarding a deck decreases that value of selecting that deck). In the context of the current task, on the first free choice trial, acquired information is thought to have a negative drive- it is beneficial for the subject to explore additional options that they never or less experienced during the forced- choice task. Therefore, in the kRL model, we model the impact of information as negative: after each forced-choice task, kRL subtracts the information gained *I*_*t*,*j*_(*c*) to the expected reward value *Q*_*t*+1_,_*j*_(*c*):

5 $${V}_{t,j}(c)={Q}_{t+1,j}(c)-{I}_{t,j}(c)\ast \omega $$

*V*_*t*,*j*_(*c*) is the final value of the deck *c*. *ω* is a weighting parameter that determines the degree by which the model integrates information in choice value *V*_*t*,*j*_(*c*). Information accumulated during the forced-choice task scales values *V*_*t*,*j*_(*c*) so that increasing the number of observations of one option decreases its final value. In other words, when one option is over-selected *I*_*t*,*j*_(*c*) becomes larger resulting in lower *V*_*t*,*j*_(*c*) On the contrary, if one option is never-selected *I*_*t*,*j*_(*c*) is zero, and ${V}_{t,j}(c)={Q}_{t+1,j}(c)$. *ω* is a weighting parameter that determines the degree by which the model integrates information in choice value *V*_*t*,*j*_(*c*).

Choices are made probabilistically using the softmax rule where options with higher values *V*_*t*,*j*_(*c*) result in higher probability to be selected:

6 $$P(c/{V}_{t,j}({c}_{i}))\,=\frac{\exp \,(\beta \times {V}_{t,j}(c))}{{\sum }_{i}\exp \,(\exp \,\beta \times {V}_{t,j}({c}_{i}))}$$

#### Model predictions

In order to derive behavioural predictions, we simulated the sRL and kRL models performing our sequential gambling-task (forced-choice task + free-choice task). For the purpose of the simulation, we drew random values for model parameters *α*]0,1], *β*]0,20], *ω*]0,20] at every simulation. Both models were simulated 80 times. As the difference between the two models related specifically to information computation, we examined their predictions principally in the Information condition, even though the model was simulated in all experimental conditions. Predictions for the equal information conditions are also reported considering their relevance for the behavioural analysis.

#### Prediction 1: Directed Exploration vs. Exploitation

In the information manipulation condition, in which decks are sampled unevenly during the forced-choice task, the kRL model predicts more frequent selection of the 0seen deck compared to the other two decks. Because kRL integrates information into a composite value estimate, the probability of choosing the 0seen deck is higher compared to exploitation (i.e., choosing the deck with the highest average of points obtained in the forced-choice task) or random exploration (i.e., choosing the experienced deck with lower point average) (Fig. 2a) (both p < 10^−3^). Under kRL, the value of an option is adjusted by the amount of information gained during the forced-choice task. Increasing the number of observations of a deck decreases its value *V*_*t*,*j*_(*c*) and thus its probability of being selected. Because the 0seen deck has not been observed, there is no penalty associated with it, resulting in a higher *V*_*t*,*j*_(*c*) and higher probability of selecting the 0seen over the 4seen and 2seen options.

The sRL model, on the other hand, predicts a higher probability of selecting the option with the highest expected reward (exploitation), compared to the other two options (Fig. 2a, both *p* < 10^−2^). Because in sRL information does not influence the value of observed options, the tendency to select 0seen is reduced and, the probability of selecting highly-rewarded and well-known options increases. Thus, under the kRL model, humans are expected to preferentially select options in order to gain more information, while the sRL model suggests that humans should select the option with the highest expected payoff.

#### Prediction 2: Reward Context

Both the sRL and kRL models predict reward context-dependent changes in directed exploration (Fig. 2 b, both *p* < 10^−3^). In the high-reward context (generative mean of all decks is 50) the expected reward *Q*_*t*+1_,_*j*_(*c*) from experienced decks is higher than the prior expectation of the decks *Q*_0_. The long-run value of *Q*_0_ is around the mean of the two Gaussian distributions (~40 points). In the High Reward context, *Q*_*t*+1_,_*j*_(*c*) increases for decks that are sampled during the forced-choice component of the task, while the expected value of the 0seen decks is not updated, ${Q}_{t+1,j}(0seen)={Q}_{0}\,$.

As a consequence, the expected value of the 0seen deck is lower than the expected value of the other two decks, and the probability to select the 0seen deck decreases. Conversely, in the Low Reward context, the Q values for decks sampled during the forced-choice task decrease below the prior Q value, with the result that the Q value for the 0seen deck tends to be higher, resulting in an increased likelihood of selecting the 0seen option. However, the two models draw different predictions concerning the degree by which the probability of selecting the 0seen deck. Because kRL integrates information with Q values during choice, the choice value *V*_*t*,*j*_(*c*) the probability of selecting the 0seen deck is higher relative to the 2seen and 4seen options in both reward contexts (Fig. 2b, all *p* < 10^−3^). In contrast, while the sRL predicts a higher probability of selecting the 0seen option in the Low Reward context (similar to kRL), it also predicts a higher probability of selecting the option with the highest Q value in the High Reward Context (Fig. 2b, both *p* < 10^−3^). Additionally, we also looked at the effect of reward contexts on random exploration in the equal information condition. Both models predict participants to be more likely to engage in random exploration in the High Reward context compared to Low Reward context (Fig. 2c, both *p* < 10^−3^).

### Model fitting and model comparison

To decide which model better explains participants’ behaviour during the task, we collected trial-by-trial participants’ choices to fit the free parameters *α*, *β*, (sRL & kRL) and *ω* (kRL) of each model (Table S1). Parameters were fit using the first free-choice trials, as in^2^ and all free-choice trials as reported in the supplementary material. During the fitting procedure, the negative log likelihood - $\sum _{1=1}^{j=128}\,\mathrm{log}({P}_{j}(c))\,\,$for each participant under each model was computed. The negative log likelihood was then minimized to obtain an estimate of the parameters for each participant under both models using MATLAB and Statistics Toolbox Release 2015b unconstrained optimization function *fminsearch*. The resulting negative-log likelihoods were used to compute the model evidence (or the log model evidence - the probability of obtaining the observed data given a particular model). We adopted an approximation to the (log) model evidence, namely Bayesian Information Criterion (BIC^18^) and Akaike Information Criterion (AIC^19^). Model evidence was used as a comparing estimate of the accuracy of the two models in describing participants’ performance on our task.

### Statistical analysis

Statistical analysis was performed using RStudio (https://www.rstudio.com/), functions and packages adopted are reported in the results section. To determine how reward and information affected strategy selection, we conducted repeated measure ANOVA. When violations of parametric tests were indicated, non-parametric tests were performed. All statistical tests were two-tailed. Multiple tests were corrected by Bonferroni correction and a *p*-value of <0.05 was considered significant.

### Data availability

The data that support the findings of this study are available from the corresponding author upon request.

## Electronic supplementary material

Supplementary Material
