# Publisher Correction: Learning the value of information and reward over time when solving exploration-exploitation problems

**DOI:** 10.1038/s41598-018-22685-z

**Published:** 2018-03-06

**Authors:** Irene Cogliati Dezza, Angela J. Yu, Axel Cleeremans, William Alexander

**Affiliations:** 1 Centre for Research in Cognition & Neurosciences (CRCN), Université Libre de Bruxelles, Brussels, Belgium; 2 Department of Experimental Psychology, Ghent University, Gent, Belgium; 3 Department of Cognitive Science, University of California San Diego, La Jolla, CA United States

Correction to: *Scientific Reports* 10.1038/s41598-017-17237-w, published online 05 December 2017

In the original version of this Article, the author Irene Cogliati Dezza was incorrectly indexed. This error has now been corrected.

