# Supplementary material for: Learning the value of information and reward over time when solving exploration-exploitation problems
Source: Sci Rep. 2017 Dec 5;7:16919. doi: 10.1038/s41598-017-17237-w (PMC5717252; doi:10.1038/s41598-017-17237-w)
Supplement: Supplementary file 1 — Supplementary Material [file 41598_2017_17237_MOESM1_ESM.pdf]

Supplementary Material: Learning the value of information and reward over time when solving exploration-exploitation problems

Irene Cogliati Dezza[1,*], Angela J. Yu[3], Axel Cleeremans[1], William Alexander[2]

[1] Centre for Research in Cognition & Neurosciences (CRCN), Université Libre de Bruxelles, Brussels, Belgium

[2] Department of Experimental Psychology, Ghent University, Gent, Belgium

[3] Department of Cognitive Science, University of California San Diego, La Jolla, CA, US

[*]icogliat@ulb.ac.be

Supplementary Results

Performance

To investigate whether participants were actively engaged in the task (i.e., learning reward outcomes to increase their total gain) we investigated the degree by which participants played strategically during the task maximizing their total gain. To do so, we computed the overall-exploitative choices (i.e. choosing the deck with the highest averaged of points obtained in the previous trials) made by participants during the entire free-choice task. All participants scored above chance level set at 33%. A Wilcoxon Signed Rank Test on the average value of subjects' overall-exploitative choices revealed a significant difference between exploitative choices (M = 0.650, SD = 0.060) and chance level, $p < 10^{-7}$, indicating that participants played strategically during the task. We also asked whether participants' performance was affected by the 4 conditions adopted in the task. To examine choice behaviour across conditions, a Friedman Rank Sum Test was conducted on overall-exploitative choices under Baseline, Reward, Information and Mixed conditions and rendered a X^2 (3, N=21) = 49.971, $p < 10^{-11}$. Pairwise comparisons using Wilcoxon Signed Rank Test revealed a significant increase in overall-exploitative choices in the Reward condition compared to Baseline, Information and Mixed condition, all p values $< 10^{-5}$. Overall-exploitative choices in the Mixed condition were greater than those during Information and Baseline conditions, all p values $< 10^{-3}$. Participants' performance was compromised in situations containing equal generative means, which make the identification of the best option challenging.

After fitting the kRL model to the first free-choice trials (see method section for details), we found that certain participants had lower values of α possible suggesting these participants were learning almost nothing during the forced-choice task (Table S1). We investigated the reason behind this apparent 'absence of learning' in certain participants during the forced-choice task. We calculated Pearson correlations between learning rate α and the probability to exploit in the first free-choice trial of the unequal information condition and equal information condition. The results showed a positive correlation between exploitation and α in the unequal information condition (r = 0.692, n = 21, $p < 10^{-3}$), however no

correlation was found in the equal information condition, $p = 0.202$. These results seem to suggest that because α affects only reward values, participants that were more prone to directed explore the environment tends to have lower α value. Indeed, a negative correlation was observed between the probability to directed explore the environment and participants' learning rate α, r = -0.563, n = 21, $p = 0.008$. Overall, these results indicate that, in those participants, lower values of α seem not to refer to an absence of learning during the forced-choice task, but rather to a preference profile toward unknown options.

Qualitative model comparison analysis

Overall behaviour in the unequal information condition

To inspect participants' behaviour overall in the unequal information condition, we computed directed exploration, random exploration and exploitation in the first free choice trials of the unequal (Information + Mixed) information condition. Trials were classified as directed exploratory when participants chose the option that was never selected during forced-choice trials, exploitative when participants chose the deck with the highest average of points and random exploratory when the classification did not meet the previous criteria. Averaged values entered into Friedman Rank Sum Test. Results revealed an effect of information on decision strategies X^2 (2, N=21) = 35.88, $p < 10^{-8}$ (Figure S1). Pairwise comparison using Wilcoxon Signed Rank Test showed significant differences between all comparisons with all p values $< 10^{-2}$, indicating that under the manipulation of information participants chose significantly more the most informative options (directed-exploratory strategy; M = 0.646, SD = 0.179) to the detriment of the options associated with the highest amount of points (exploitation; M = 0.283, SD = 0.152) and the random exploratory options (M = 0.086, SD = 0.069). Similar pattern was obtained when we computed the decision strategies in the Mixed condition only, X^2 (2, N=21) = 32.268, $p < 10^{-8}$.

Insert Figure S1

Reward context and utility vs. cost trade-off

In the analysis on the effect of reward context on decision strategies we observed that the two exploratory strategies showed diametrically opposite effect following changes in reward context as predicted by the two models. To better understand why random exploration was higher in the High Reward context, we conducted an additional analysis in the equal information condition (Baseline + Reward condition). We hypothesized that the reason might be due to an effect of utility vs. cost of staying/switching among options induced by the reward context. In other words, staying with the same option in low reward context where the majority of options are poor in value is more advantageous than switching among options because the utility of the switch might be lower than its cost (because all the options can be expected to be bad). In contrast, switching in the high reward context where the majority of the options are associated with higher payoffs might be more advantageous and the utility of the switch might overcome its cost. Additionally, this effect might also depend on the relative reward obtained on the trial preceding the switch (whether it was better or worse than expected). To test this possibility, we computed the probability of participants staying with the same option after the first free choice trial during the Low and High Reward condition given a positive and a negative prediction error (PE) (generated after receiving a feedback at the end of the first free-choice trial). We computed PE using the δ learning rule and the estimated parameters for each subject using kRL. We conducted a non-parametric 2 (context: High Reward, Low Reward) by 2 (Prediction Error: PE-positive, PE-negative) ANOVA on the probability of staying with the same option after the first free choice trial. The results showed an effect of PE $F(1,60) = 247.4$, $p < 10^{-15}$, and effect of PE X Reward Context $F(1,60) = 8.8$, $p = 0.004$) (Figure S2), whereas a general effect of context did not reach significance, $p = 0.549$. Post-hoc comparison indicated an increment in the probability of stay given PE-negative in the Low Reward (M = 0.143, SD = 0.105) compared to High Reward (M = 0.044, SD = 0.056), $p = 0.0007$, and a marginally increase in the probability of stay given PE-positive in High Reward (M = 0.618, SD = 0.1929) compared to Low Reward (M = 0.538, SD = 0.12), $p = 0.055$. These results showed that even if the reward obtained was worse than expected, staying with the

same option was preferred when the majority of the options are associated with lower payoff compared to high reward context. These results suggest that a utility/cost trade-off might interact with the decision to explore the environment under different reward context.

Insert Figure S2

Quantitative model comparison analysis

Simulation with fitted parameters

After fitting data with both kRL and sRL, we simulated behavioural output using the fitted parameter (Figure S3). The analysis revealed a similar pattern observed during the simulation with random parameters. KRL often selects 0seen deck compared to the other two decks ($p < 10^{-3}$), and this pattern was absent in sRL ($p > 0.05$). Concerning the effect of reward context, both models showed a decrease in directing exploring the 0seen deck as a function of reward context ($p < 10^{-3}$). As predicted, when simulating kRL model the probability of selecting the 0seen deck is higher relative to the other two options in both reward contexts (all $p < 10^{-3}$), whereas with sRL model the probability of selecting the 0seen deck is lower compared to the probability to select the exploitative deck in High Reward Context ($p < 10^{-3}$).

Insert Figure S3

Correlations among model parameters

After fitting the kRL model (Table S1), we observed correlations between model parameters. Specifically, we observed a negative correlation between the learning rate α and the softmax inverse temperature parameter β, r = -0.587, n = 21, $p = 0.005$, and a negative correlation between the inverse temperature parameter β and the information parameter ω, r = -0.723, n = 21, $p < 10^{-3}$. The first correlation is a standard correlation found in the literature and is due to the inability of decision tasks like ours to dissociated the two parameters [1]. The second correlation might either reflect an interaction between directed (higher ω) and random (lower β) exploration previously reported in previous studies [2] or an artefact of the fitting procedure. We better investigated this point by fitting the synthetic data we obtained

when simulating kRL with random parameters (see Methods). Pearson correlation did not reveal any correlation between α and β, $p = 0.361$ and β and ω, $p = 0.117$. This result seems to confirm that both exploratory strategies share a common exploratory drive as proposed in previous research [2].

Fitting over all free-choice trials

Our fitting procedure was also conducted on all free-choice trials of the gambling-task to test whether the kRL model might extend to all choices performed by participants. We conducted model comparisons between sRL and kRL using a Wilcoxon Signed Rank Test. The results showed that kRL model ($BIC_{krl} = 791.092$) best represented participants' data compared to sRL ($BIC_{sRL} = 809.064$), Z= 3.597, $p < 10^{-3}$, confirming the results obtained with the previous fit.

Priors over Q values

Here, we investigated whether participants were using a static global estimate of the expected reward values for each deck, as assumed by our implementation of kRL, or if estimates of the expected value of each deck was updated throughout the task. We compared the version of the kRL model described in the main text (Figure 1) to a new version of kRL that uses a δ learning rule [3] to update initial value estimates, Q_0, at the beginning of each new game:

$$Q_{0,j}(c) = Q_{0,j-1}(c) + \alpha_P \times \left(Q_{final,j-1}(c) - Q_{0,j-1}(c)\right) \quad \text{(S1)}$$

where $Q_{0,j-1}(c)$ is the initial Q_0 value of the previous game of option c and $Q_{final,j-1}(c)$ the expected value of option c in the last trial of the previous game. α_p is the learning rate for the prior that describes the degree by which the new prior is updated by the new expected value $Q_{final,j-1}$. Here, the $Q_{0,j}(c)$ oscillated around the global estimate Q_0 depending on actual rewards experienced by participants during each game. We fitted this version of the kRL model to the choices made by participants in the first free-choice trials. A paired sampled t-test showed higher BIC for kRL model that was using the above updating rule ($BIC_{kRL} = 166.596$) compared to kRL with fix rule ($BIC_{kRL} = 163.978$), $t(20)=2.625$, $p = 0.016$. The result seems to suggest that participants were using a global estimate of the expected reward values for each deck instead of learning it on a trial basis.

Learning dynamics

After evaluating whether participants were learning reward outcomes (using a global estimate of Q prior) and accumulating information during the decision process, we questioned the type of learning that took place during the task. We assumed that participants used the δ learning rule to update reward expectations on each trial. This assumption was based on the vast literature on the use of this rule by humans when updating expected reward value [4]. However, because participants need only to track observed outcomes during the forced-choice task and make own choices at the end of it, it might be possible that they estimated an average valued of reward as predicted by Wilson et al. Furthermore, we also assumed that the degree by which the update took place was constant throughout the experiment; in our model fits, we assumed a constant learning rate. However, participants might integrate new reward outcomes differently from one trial to another [5]. We investigated these two points comparing the kRL model to a linear model (LM, with two free parameters) that linearly scaled reward expectation as kRL. However, the linear model computed reward expectation as average values of observed reward as follows:

$$Y_{t=6,j}(c) = \left(\frac{prior}{n+1}\right) + \left(\frac{n}{n+1} * \mu R(c)_{t=1:6,j}\right) - \left(\beta_I * \sum_{1}^{t=6} i_{t=1:6,j}\right) \qquad \text{(S2)}$$

where $\frac{n}{n+1}$ is a weight term that changes as a function of the number of observations *n*, and *prior* is the prior estimate of the expected value at the beginning of each game (which is assumed to be fixed as in kRL). The first two components of the model thus vary the influence of prior information and recent information based on the number of observations; with no observations, the estimated value of each option is entirely based on the prior, while with a high number of observations, the estimated value of each option depends primarily on the observed rewards. $\mu R(c)$ is the mean of observed outcomes during the forced-choice task, and β_I is the weight of information. The model makes choices the softmax rule as showed in equation (6), where choice values are replaced by $Y_{t=6,j}(c)$. We conducted the optimization procedure as described in the methods section. We then computed the BIC related to the two models and run a Wilcoxon Signed Rank Test. Results showed that kRL model (BIC_{kRL} = 163.978) best represented participants' data compared to LM (BIC_{LM} = 172.063), Z=3.215, p = 0.0006. This result suggests that participants adopted a

δ learning rule with a fixed learning rate as opposed to estimating the reward using a long-term average of observed outcomes. This is consistent with our previous findings that humans weigh recent observations more than distant ones in reward learning in the bandit task [6].

Parametric modulation of information

After clarifying the learning rule adopted by participants, we investigated whether participants were integrating information from the forced-choice task as function of the number of observations (as predicted by kRL model) or as information bonus equally weighting less seen options (i.e. 2seen and 0seen deck- similar to [7]). To do so, we implement a constant-information model (ckRL) were equation (4) was modified such that:

$$1I_{t,j}(c) = \begin{cases} 1, & choice = 0/2seen \\ 4, & choice = 4seen \end{cases}$$

CkRL computes choice values $V_{t,j}(c)$ as in equation (5) and choices are made using the softmax function of equation (6). We fitted ckRL to participants' choices of first free-choice trial. Model comparison between ckRL and kRL revealed that kRL model ($BIC_{krl} = 163.978$) best represented participants' data compared to ckRL ($BIC_{ckRL} = 233.343$), $t(20) = 6.399$, $p < 10^{-5}$. This result seems to suggest that participants integrated information in a parametric fashion and not as constant information bonus among less seen alternatives.

Supplementary References

1. Daw, N.D., *Trial-by-trial data analysis using computational models*, in *Affect, Learning and Decision Making, Attention and Performance XXIII*, E.A. Phelps, T.W. Robbins, and M. Delgado, Editors. 2009, Oxford University Press: New York.
2. Zajkowski, W.K., M. Kossut, and R.C. Wilson, *A causal role for right frontopolar cortex in directed, but not random, exploration.* Elife, 2017. **6**.
3. Rescorla, R.A. and A.R. Wagner, *A theory of Pavlovian conditioning: Variations in the effectiveness of reinforcement and nonreinforcement.* Classical conditioning: Current research and theory, 1972: p. 64-99.
4. Preuschoff, K. and P. Bossaerts, *Adding prediction risk to the theory of reward learning.* Ann N Y Acad Sci, 2007. **1104**: p. 135-46.
5. Behrens, T.E., et al., *Learning the value of information in an uncertain world.* Nat Neurosci, 2007. **10**(9): p. 1214-21.
6. Zhang, S. and A.J. Yu. *Forgetful Bayes and myopic planning: Human learning and decision-making in a bandit setting*. in *Advances in Neural Information Processing Systems 26*. 2013. MIT Press.
7. Wilson, R.C., et al., *Humans use directed and random exploration to solve the explore-exploit dilemma.* J Exp Psychol Gen, 2014. **143**(6): p. 2074-81.

Supplementary Figure Caption

Figure S1 *Overall behaviour.* Participants' decision strategies were computed in the unequal information condition (Information + Mix condition) - when information concerning the 3 decks was not always available. Participants were more likely to directed explore in the first trial of the free-choice task compare to exploit and random explore as shown for the Information condition and predicted by kRL.

Figure S2 *Positive and negative prediction error*. In the equal information condition (when information concerning the 3 decks was always available), making choices in Low Reward context (when the generative mean was set to 30 points) increased the probability of stay with the same option after a negative prediction error compared to the high reward context (when the generative mean was set to 50 points). Probability of stay with the same option after a positive prediction did not differ between Low and High Reward context.

Figure S3 *Model simulation with fitted parameters*. Simulating both kRL and sRL model with the parameter estimated by the fitting procedure revealed similar pattern observed with the simulation using random parameters. a) kRL (left frames) shows a higher probability of choosing the most informative/never experienced option (i.e., 0seen) compared to exploitative option, whereas in sRL (right frames) this difference disappears. b) Both models show a decrement in directed exploration in high reward contexts compared to low reward contexts. However, in the high reward context, kRL still chooses more often 0seen options compared to exploitation (left frames), whereas sRL chooses more often exploitative options (right).

Supplementary Figure

Figure S1

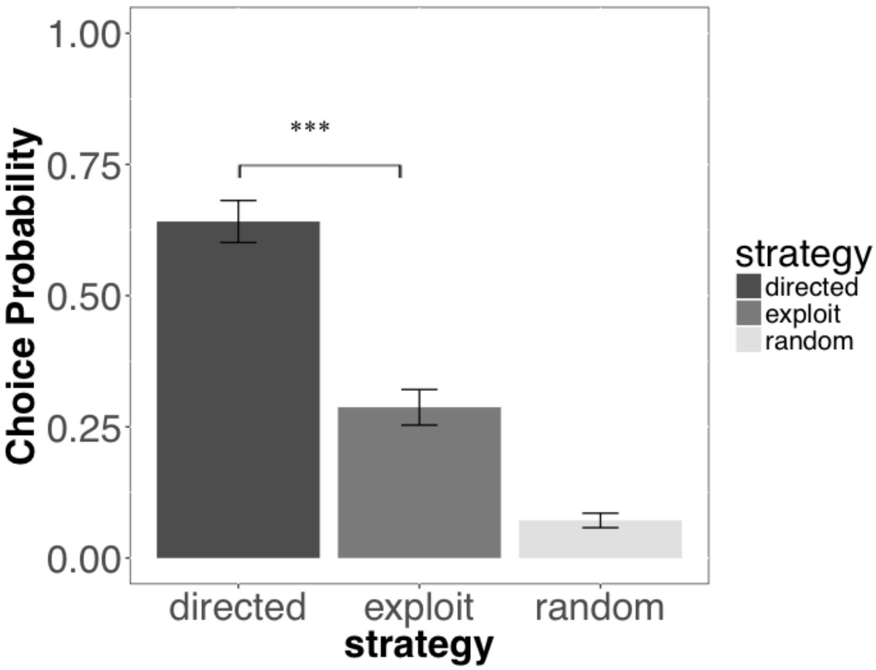

Figure S2

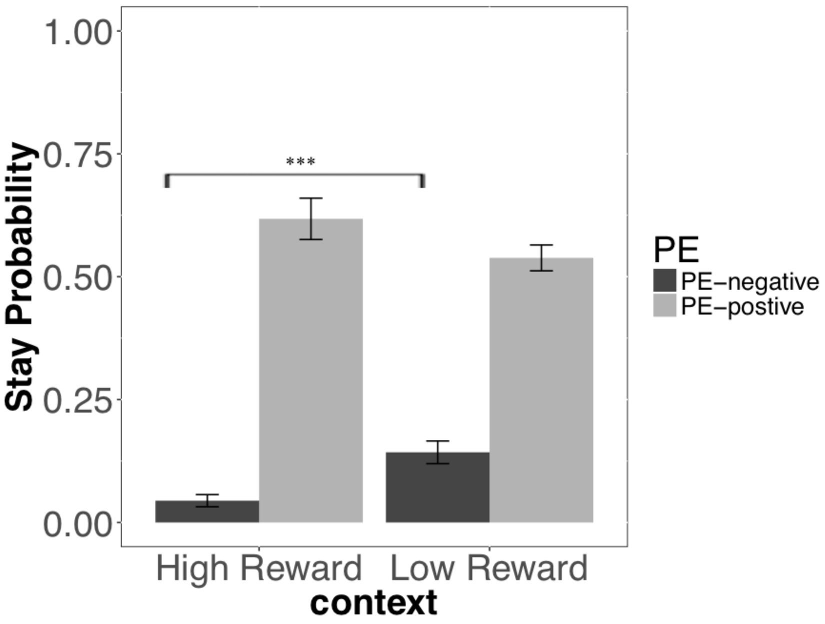

Figure S3

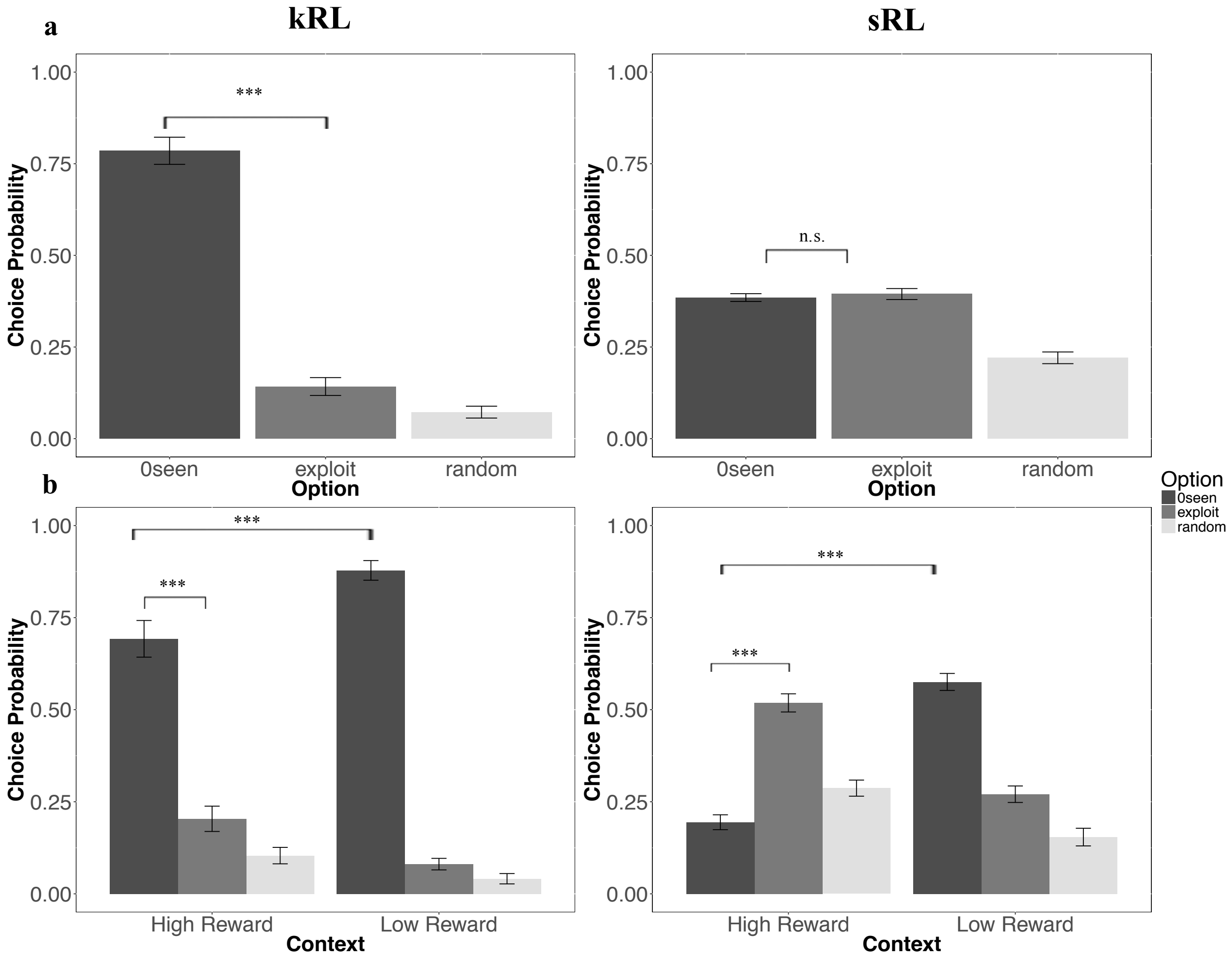
a
kRL
sRL
***
n.s.
1.00
0.75
0.50
0.25
0.00
Choice Probability
0seen
exploit
random
Option
b
***
***
***
***
High Reward
Low Reward
Context
Option
0seen
exploit
random

Supplementary Table Caption

Table S1 *Model fit results*. Estimated true parameters for each subject given sRL model and kRL model. Parameters were fit using the first free-choice trials. Group average of estimated parameters are also reported. Standard deviations are reported within parenthesis.

Supplementary Table

Table S1

	sRL		kRL		
Participants	α	β	α	β	ω
Number1	0.588	0.041	0.006	7.031	0.191
Number2	0.659	0.075	0.189	0.364	3.681
Number3	0.548	0.034	0.004	5.646	0.149
Number4	0.719	0.112	0.320	0.278	3.688
Number5	1.011	0.028	0.041	3.144	1.769
Number6	0.592	0.109	0.232	0.348	3.268
Number7	0.922	0.020	0.013	4.255	0.672
Number8	0.652	0.092	0.414	0.168	4.193
Number9	0.914	0.048	0.129	0.488	3.448
Number10	1.022	0.031	0.066	1.316	2.532
Number11	0.874	0.035	0.132	0.784	4.473
Number12	0.928	0.050	0.207	0.309	4.902
Number13	0.733	0.134	0.687	0.142	1.108
Number14	0.878	0.044	0.163	0.327	4.332
Number15	0.346	0.124	0.046	1.703	0.889
Number16	0.635	0.075	0.180	0.417	3.451
Number17	0.620	0.080	0.087	2.097	2.063
Number18	0.063	0.145	0.036	0.326	0.690
Number19	1.031	0.035	0.162	0.320	5.165
Number20	0.737	0.029	0.004	3.955	0.111
Number21	0.714	0.039	0.008	2.706	0.185
Total	0.723<br>(0.235)	0.066<br>(0.039)	0.149<br>(0.166)	1.720<br>(2.012)	2.427<br>(1.759)
